# Modeling Postoperative Pathologic Ileus in Mice: A Simplified and Translational Approach

**DOI:** 10.1111/nmo.70157

**Published:** 2025-09-22

**Authors:** Romain Gauthier, Julie Thevenin, Thibault Planchamp, Téo Berthon, Perrine Rousset, Shuai Wang, Astrid Canivet, Claude Knauf, Nathalie Vergnolle, Etienne Buscail, Céline Deraison

**Affiliations:** ^1^ IRSD, University of Toulouse, INSERM, INRAE, ENVT, Université Toulouse III—Paul Sabatier (UPS) Toulouse France; ^2^ Department of Digestive Surgery Toulouse University Hospital Toulouse France

**Keywords:** chirurgical procedure, gut motility, inflammation, intestinal transit, postoperative ileus

## Abstract

**Background:**

Postoperative Ileus (POI) is an iatrogenic complication characterized by a temporary paralysis of gastrointestinal transit, leading to food intolerance, nausea, vomiting, and thus prolonged hospitalization. The severity of POI is influenced by surgical trauma, particularly in intestinal surgeries, which have a high complication rate. To date, no animal model has precisely replicated POI in the context of digestive sutures or anastomoses, procedures common in human digestive resections.

**Methods:**

To induce POI, mice underwent different surgeries. The surgical procedure involved a midline laparotomy, externalization of the small intestine following external manipulation through moistened cotton applicators, mimicking the surgeon's action when searching for an intestinal lesion. An ileo‐ileal anastomosis was also performed to ensure relevance to human surgical intervention. Intestinal transit was measured by assessing gastric emptying, gastrointestinal transit time (gavage with charcoal, fecal output), and gut motility (isotonic contraction). Postoperative inflammation is assessed in different tissue layers and areas of theintestine.

**Key Results:**

The externalization of the small intestine with caecum and manipulation for 10 min induced a pathological postoperative ileus. This simple gesture induced a decrease in intestinal transit comparable to the surgical intervention, ileo‐ileal anastomosis. The model showed decreased gastric emptying and reduced ileal muscle contraction, accompanied by neutrophil and monocyte/macrophage infiltration in the external muscularis.

**Conclusions and Inferences:**

The developed procedure enables inducing postoperative ileus in mice in a very simple and reproducible way that does not require any specific equipment, mimics clinical practice, and reproduces traits of human pathology.

## Graphical abstract

Simple manipulation of the small intestine for 10 min induces pathological postoperative ileus, as evidenced by reduced gastrointestinal transit and inflammation in the muscular layer.

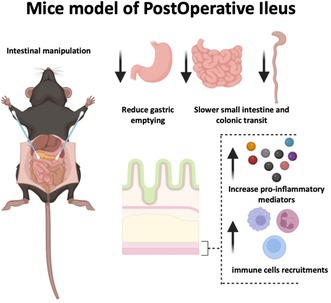

**Summary**

- A simple intestinal manipulation that mimics the surgeon's action causes gastrointestinal dysmotility lasting up to 4 days post‐surgery, leading to a pathogenic postoperative ileus.
- The severity of this model is comparable to that observed in clinically relevant human intestinal anastomosis surgery.
- This simple manipulation induces inflammation in the muscular layer, a hallmark of postoperative ileus.

## 1 Introduction

Postoperative ileus (POI) is a common iatrogenic condition associated with abdominal surgery, characterized by a prolonged absence of gastrointestinal (GI) transit in response to surgical intervention. Clinically, patients suffering from POI manifest as intolerance to oral intake, abdominal distension, and impaired GI propulsion, without evidence of mechanical obstruction [1]. It is widely accepted that a transient, self‐limiting form of POI represents a normal physiological response to abdominal surgery, typically resolving without significant complications. However, when POI becomes pathologic and prolonged, it contributes to increased patient morbidity, discomfort, and dissatisfaction, leading to extended hospital stays and higher healthcare costs [2].

The severity and duration of POI are largely determined by the extent of surgical trauma, with intestinal surgery being particularly associated with a high incidence of complications. Notably, patients experiencing POI face a complication rate as high as 60%, with 20% requiring admission to intensive care. Even with the implementation of Enhanced Recovery After Surgery (ERAS) protocols, POI continues to pose a significant clinical challenge, particularly in colorectal surgery, where its incidence can reach 20% [3].

Economic analyses highlight the substantial financial burden of POI, with U.S. healthcare expenditures for its management rising from $7.1 billion in 2001 to $12.3 billion in 2011 [2]. Direct costs per patient increased by approximately €8233, representing a more than 60% increase per hospitalization [4]. Therefore, the early restoration of postoperative intestinal function is critical not only for improving clinical outcomes and quality of life, particularly in gastrointestinal surgeries, but also for reducing hospital length of stay, increasing hospital bed turnover, and conserving medical resources. Although various strategies have been proposed to mitigate the duration of POI, their efficacy remains limited, and no single treatment has been proven to be definitively superior [5].

The precise mechanisms underlying POI are not fully understood. Surgical opening of the peritoneal cavity and manipulation of the digestive tract initiate a biphasic response [6]. The initial “neurogenic phase,” mediated by the activation of the sympathetic nervous system, occurs within the first few hours post‐surgery [7, 8]. This is followed by a later, inflammation‐driven phase, beginning 3–4 h postoperatively and persisting for several days, which is believed to be the most clinically relevant [9, 10]. No animal model to date has precisely replicated POI in the context of digestive sutures or anastomoses, procedures that are routinely performed in humans following digestive resections. This development induced by intra‐abdominal surgery was designed to closely replicate the effects of abdominal surgery on the digestive system and to support a standardized method for studying POI in mice. This accurate model will allow us to investigate the underlying mechanisms of POI and evaluate new therapeutic strategies.

## 2 Materials and Methods

### 2.1 Animals

Mice aged 8–12 weeks, male wild types (C57BL/6J), were purchased from Janvier. All animals were maintained on a 12‐h light–dark cycle with free access to water and food, except for an 18‐h fasting period prior to charcoal administration for the measurement of gastric function and small intestine transit as well as fecal output. The Animal Care and Ethics Committee of US006/CREFRE (APAFIS #202011201302875) approved all experimental procedures, and they were performed following the guide for the care and use of laboratory animals of the European Council.

CX3CR1‐Cre^ERT2^YFP^+/−^ (mice express a Cre‐ERT2 fusion protein and Yellow Fluorescent Protein (YFP) under CX3CR1 promotor) were also used in this study to visualize macrophages and monocytes in intestinal tissue.

### 2.2 Operative Procedure

Before surgery, animals were anesthetized by intraperitoneal injection of a solution of Ketamine/Xylazine (100/10 mg/kg) with subcutaneous administration of buprenorphine (0.01 mg/kg) for analgesia. Mice were subjected to different types of surgery with the aim of inducing postoperative ileus:

The surgical procedure consisted of a midline laparotomy followed by externalization of the small intestine (SI Ext.), externalization of the cecum (SI + caecum Ext.), manipulation of the small intestine for 2 min (SI + caecum Ext. + IM2′), or manipulation of the small intestine for 10 min using moist cotton applicators, mimicking the surgeon's action when searching for an intestinal lesion (SI + caecum Ext. + IM10′). Mice undergoing laparotomy alone (Sham) represented the control group. Finally, steady‐state mice represented mice without any anesthesia and surgery. A summary of all surgical procedures is provided in Table S1. It should be noted that the intestine was not compressed, but manipulated with the aim of unwinding and preserving the mesentery to be as close as possible to human surgical practice.

The small intestine was regularly hydrated with NaCl to prevent dehydration during surgery. Afterward, it was repositioned into the abdominal cavity, and the incision was closed by performing two X‐shaped separate sutures on the peritoneal and cutaneous layers. Mice were kept in contact with a heating pad during surgery and until they woke up from anesthesia (maintained at 37°C).

An ileo‐ileal anastomosis was conducted. Following the externalization of the intestine, intestinal manipulation during 5 min was realized. After, a complete transverse section of the terminal ileum was performed, 1 cm proximal to the ileocecal valve, while preserving the mesentery. Subsequently, a suture comprising four simple equidistant points between the two ileal segments was meticulously executed, ensuring that the knots are strategically positioned at the four cardinal points.

The surgical procedures resulted in no deaths or major surgical complications (including hemorrhage, peritonitis or perforation). All animals appeared healthy, with early post‐surgical fluid intake and normal behavior.

### 2.3 Measurement of Gastrointestinal Transit

The mice were fasted for 18 h prior to the measurement of gastric and small intestine motor functions. Gastric emptying, small intestine transit, and duodenum diameter were assessed after surgery by oral gavage of 500 μL with a black solution (charcoal 5% w/v and acacia gum 10% w/v (all from Sigma, Saint‐Quentin‐Fallavier)). Twenty minutes after gavage, mice were euthanized, and the digestive tract was removed. Gastric emptying was determined by measuring the distance from the pylorus to the beginning of charcoal migration, with results expressed in centimeters. Complete gastric emptying was defined when the distance was > 0 cm. Thus, small intestine transit was determined by the distance from migratory charcoal, normalized by the length of the small intestine (from pylorus to ileocecal valve). In order to be as close as possible to human pathophysiology, measurements of gastric emptying and intestinal transit were taken at 24, 48, 72, and 96 h after surgery [11]. Fecal output was recorded every 24 h, beginning immediately after surgery and continuing for 96 h. The stool count was determined by dividing the total number of feces produced during this period by the number of mice housed in the same cage. The diameter of the duodenum was measured using calipers. These measurements were made to be as close as possible to the definition of the POI proposed in 2018 and defined by the IFEED score [11].

The integrity of all ileo‐ileal anastomoses was assessed the following day after sacrifice, meticulously examining for any signs of fistula and ensuring the patency of the anastomosis.

### 2.4 Measurement of Intestinal Contractions

After dissection, duodenum, jejunum, ileum, and colon segments were washed in Krebs‐Ringer bicarbonate/glucose buffer (pH 7.4) in 95% O_2_ and 5% CO_2_. Intestinal segments were then incubated in oxygenated Krebs‐Ringer solution for 30 min at 37°C, attached to the isotonic transducer (MLT7006 Isotonic Transducer, Hugo Basile, Comerio, Italy), and immersed in an organ bath of the same medium maintained at 37°C. The load applied to the lever was 1 g (10 mN). Isotonic contractions were recorded on Labchart software (AD Instruments) following the transducer displacement. After attaching the intestinal segments, basal contractions were recorded for 15 min. The amplitude of muscle contractions was presented as a percentage relative to the basal response, whilst the frequency of contractions was presented as the number of contractions per minute., as previously described [12, 13].

### 2.5 Reverse Transcription—Quantitative Real Time PCR (RT‐qPCR)

The ileum and colon were emptied to remove their contents and then opened longitudinally. The longitudinal muscle myenteric plexus (LMMP) was isolated using fine forceps under a binocular magnifier, as previously described [12]. The mucosa and muscularis were stored at −80°C until processing. RNAs were isolated from the mucosa or muscularis externa of the ileum and colon using the RNeasy Kit (Qiagen). Three micrograms of RNA were utilized for cDNA synthesis with the Maxima first strand kit (Invitrogen). Quantitative PCR (qPCR) was performed using Takyon (Eurogentec) after a 1/10 dilution of cDNA synthesis on a LightCycler 480 (Roche Diagnostics).

Primers used in the study are listed in Table 1. The relative expression of target genes was normalized to the reference genes *Hprt* and *Tbp*. Subsequently, data from the muscularis and mucosa of mice subjected to intestinal manipulation (IM10′) were normalized with those from sham mice.

**TABLE 1:** Primers used for quantitative PCR.

Primer sequences for qPCR	Forward	Reverse
*mMcp1*	TTCTTTGGGACACCTGCTG	TGTTGGCTCAGCCAGATGCA
*mIl6*	CGTGGTTGTCACCAGCATCA	CTCTGCAAGAGACTTCCATCCAGT
*mIl1b*	ACCTTCCAGGATGAGGACATGAG	CATCCCATGAGTCACAGAGGATG
*mIcam1*	AGTCCGCTGTGCTTTGAGAAC	CTCTCCGGAAACGAATACACG
*mTnfa*	AATGGCCTCCCTCTCATCAG	GCTACGACGTGGGCTACAGG
*mKc*	AGCCACACTCAAGAATGGTC	GTCAGAAGCCAGCGTTCAC
*mTbp*	CAGCCTTCCACCTTATGCTC	TTGCTGCTGCTGTCTTTGTT
*mHprt*	TCAGTCAACGGGGGACATAAA	GGGGCTGTACTGCTTAACCAG

### 2.6 Histochemistry and Immunohistochemistry

#### 2.6.1 Histological Analysis

Ileal and colonic swissroll were fixed with 4% formaldehyde (VWR) for 24 h. After, samples were included in paraffin. Cross‐sections (5 μm) were stained with hematoxylin and eosin. Images were acquired with Pannoramic 250 (3Dhistech) to scan the entire ileal and colonic swissroll. Damage scoring was evaluated with a focus on epithelial/mucosal alteration, submucosal edema, and the presence of inflammatory cell infiltration.

#### 2.6.2 Immunohistochemistry and Quantification

Antigenic regeneration of cross‐sections was performed in a citrate buffer for 20 min at 95°C. Blocking and permeabilization were carried out with a blocking buffer (PBS 1× [Sigma], 1% BSA [Dutcher], and 0.5% Triton X‐100 [Sigma] for 3 h). Subsequently, samples were incubated overnight with anti‐mouse CD45 antibody in blocking buffer (1:1000; 14‐0451‐85; Thermo Fisher). The primary antibody was detected with goat anti‐rat IgG linked with Alexa Fluor 555 in blocking buffer (1:1000; A‐21434; Thermo Fisher). Samples were mounted on slides using ProLong Gold Antifade Mountant with DAPI (Invitrogen). Confocal microscope images were acquired using a Zeiss LSM710 (Carl Zeiss).

Quantification of CD45 staining in the ileal muscularis externa was performed using ImageJ. The mean fluorescence intensity from 2 to 4 images was used to assess the mean fluorescence intensity in the muscularis externa. Mean intensity were normalized with sham as baseline.

#### 2.6.3 Wholemount Staining of LMMP

Isolated LMMP tissues were fixed in 4% paraformaldehyde (VWR) for 1 h. Blocking and permeabilization were performed using a blocking buffer (PBS, 5% BSA [Dutcher], and 0.5% Triton X‐100 [Sigma]) for 1 h. Samples were then incubated for 48 h with an anti‐chicken GFP antibody diluted in blocking buffer (1:200; ab13970; Abcam). The primary antibody was detected using a goat anti‐chicken IgY secondary antibody conjugated to Alexa Fluor 633, diluted in blocking buffer (1:1000; A‐21103; Thermo Fisher). Nuclei were stained with DAPI (1 μg/mL in PBS; Sigma) for 20 min. Finally, samples were mounted on slides using ProLong Gold Antifade (Thermo Fisher Scientific). Confocal images were acquired on an Opera Phenix (Revvity) high‐content screening system. Images were acquired over the entire thickness of the sample with a 2 μm interval. A 3D analysis was performed using the Harmony software allowing detection of nuclei and YFP^+^ cells. The number of YFP^+^ cells was reported to the surface unit.

### 2.7 Quantification of CCL2 Chemokine

The ileal muscularis externa tissue was homogenized in a Precellys tube for 30 s, twice, at 5000 rpm. Proteins in the resulting supernatant were isolated by centrifugation for 5 min at 4500 rpm. The total protein quantity was determined using the Pierce Dilution‐Free Rapid Gold BCA Protein Assay (Thermo Fisher). The concentration of CCL2 chemokines in the ileal muscularis externa was measured using the Mouse CCL2/JE/MCP‐1 Quantikine ELISA Kit (Bio‐Techne) according to the manufacturer's instructions.

### 2.8 Cytofluorometric Analysis

#### 2.8.1 Cell Preparation From Ileal Muscularis Externa

The immune cells of the muscular layer of the ileum were isolated as previously described with some modifications [13]. The muscular layer of the ileum was incubated in a digestion solution containing 2 mg/mL Dispase II (Roche), 0.4 mg/mL Collagenase D (Sigma), 5% fetal calf serum (Sigma) in RPMI (Thermo Fisher) for 30 min at 37°C with slow agitation. After digestion, the cells were passed through a 70 μm cell strainer to remove undigested pieces. The cells were counted using a hemocytometer and were used in their entirety for flow cytometry.

#### 2.8.2 Cell Staining and Flow Cytometry Analysis

The cells were incubated with Live Dead Fixable Aqua (Thermofisher) in PBS for 15 min at 4°C. Then, the cells were stained in FACS buffer containing 2% fetal calf serum and 0.01% sodium azide (Sigma) with the following antibodies (from Miltenyi Biotech or Becton, Dickinson): anti‐CD45 conjugated FITC (REA737), anti‐CD11b conjugated PE‐Cy7 (REA592), anti‐F4/80 conjugated PE (REA126), anti‐Siglec‐F conjugated APC‐Cy7 (REA798), anti‐Ly6C conjugated vioblue (REA796), anti‐Ly6G conjugated Percpvio700 (REA526) and anti‐CX3CR1 conjugated AF647 (Z8‐50). The cells were washed with FACS buffer before analysis. Monocytes are CD45^+^ CD11b^+^ F4/80^−^ Siglec‐F^−^ Ly6G^−^ Ly6C^+^. Neutrophils are CD45^+^ CD11b^+^ F4/80^−^ Siglec‐F^−^ Ly6G^+^ Ly6C^low^. Eosinophils are CD45^+^ CD11b^+^ F4/80^low^ Siglec‐F^+^. Total macrophages were CD45^+^ CD11b^+^ F4/80^+^ Siglec‐F^−^. Immature macrophages in the process of differentiation express a low level of CX3CR1, while mature macrophages express a high level of CX3CR1. The gating strategy used for flow cytometry analysis was illustrated in Figure S1. Flow cytometry was performed using the Fortessa X20 cytometer (BD Biosciences). The data were then analyzed using FlowJo v10 software (BD Biosciences).

### 2.9 Data and Statistical Analysis

The data were presented as individual values and means ± SEM. Statistical analyses were performed using GraphPad Prism 10.00 [GraphPad software]. The Kruskal–Wallis test was employed for multigroup comparisons followed by a Dun's correction for multiple tests. The Mann–Whitney test was used for comparisons between two groups. The Spearman correlation coefficient was used to assess the correlation between duodenum diameter and small intestine transit. *p* Values < 0.05 were considered to be statistically significant (*p < 0.05, **p < 0.01, ***p < 0.001 and ****p < 0.0001).

## 3 Results

### 3.1 Intestinal Manipulation Induces Postoperative Ileus in Mice

**FIGURE 1:**
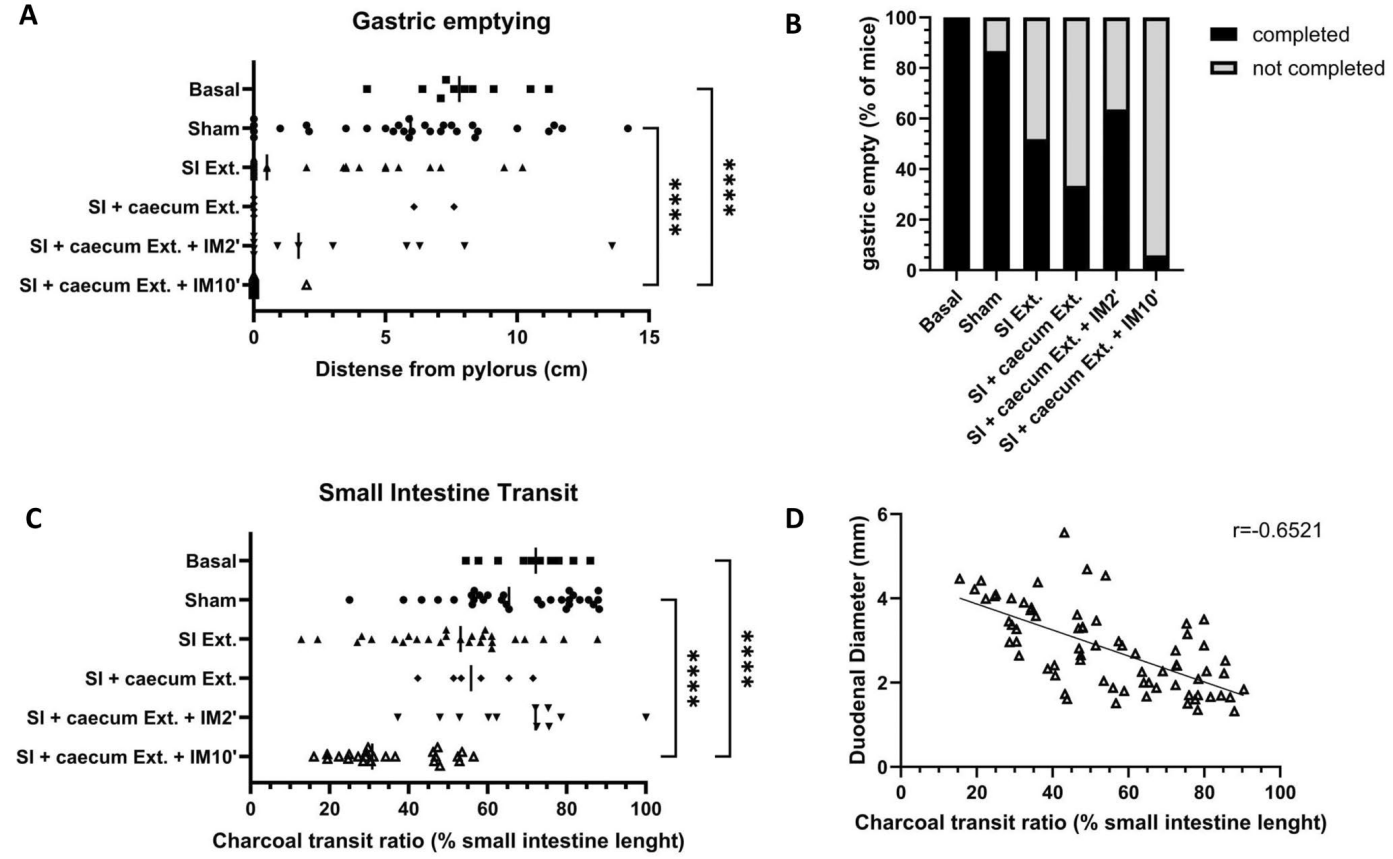
Different procedures of small intestine manipulation to develop a reproducible model of postoperative ileus in mice. For different procedures, steady‐state (Basal) and 24 h after laparotomy (Sham), small intestine externalization (SI Ext.), small intestine and cecum externalization (SI + cecum Ext.), small intestine and cecum externalization with 2 min of manipulation (SI + cecum Ext. + IM2′), or intestinal manipulation for 10 min (SI + cecum Ext. + IM10′) gastric emptying (A), index of complete gastric emptying (B), small intestine transit (C) and duodenum diameter were measured 20 min after oral gavage of charcoal. A correlation of duodenum diameter and intestinal transit was represented (D). Data are presented as individual values with means (*n* = 6–30 per group). Significance levels were determined using the Kruskal–Wallis test followed by Dunn's multiple comparisons test (A–C), and Pearson correlation was used for analysis (D): Ns (*p* > 0.05); **p < 0.01; ***p < 0.001; ****p < 0.0001.

**FIGURE 2:**
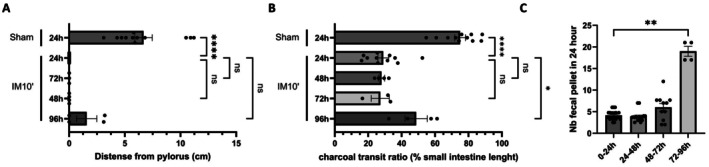
Small intestine manipulation for 10 min induced a pathological postoperative ileus in mice. Gastric emptying (A) and small intestine transit (B) were measured in mice, 24 h after laparotomy, or 24, 48, 72, or 96 h after IM10′ procedure. Fecal output (C) was recorded over a 24‐h period for 96 h following intestinal manipulation for 10 min (*n* = 3–8 per group). Data are presented as individual values with means and SEM. Significance levels were determined using the Kruskal‐Wallis test followed by Dunn's multiple comparisons test: Ns (*p* > 0.05); *p < 0.05; ****p < 0.0001.

Various surgical procedures were employed in mice to establish induce arrest of intestinal transit. After anesthesia, we performed surgeries ranging from laparotomy alone (sham) to the externalization of the small intestine with or without the cecum, followed by or without the execution of 3 intestinal round trips (IM2′), or involving intestinal manipulation during 10 min (IM10′). Gastric emptying function and small intestine transit were evaluated 24 h post‐surgery following the administration of a charcoal solution. However, externalization of the intestine with or without the cecum, along with or without performing 3 round trips using moist cotton applicators to manipulate the small intestine, induced in less than 50% of mice absence of gastric emptying and in some cases small intestine transit. However, the procedure based on 10 min of intestinal manipulation (IM10′), mimicking the surgeon's action when searching for an intestinal lesion, led to an absence of gastric emptying and a drastic slowdown in intestinal transit in all mice subjected to this intestinal manipulation (Figure 1A–C; Table S2). The charcoal covered only 36% of the small intestine after MI, whereas it covered 67% of the small intestine after laparotomy alone; intestinal transit is halved in all mice. In addition, as observed in patients with ileus showing distention of the intestinal tract [11], dilation of the duodenum was observed after 10 min of intestinal manipulation. The analysis showed a significant negative correlation between duodenal diameter and charcoal progression (Figure 1D). These findings collectively suggest that 10 min of intestinal manipulation reliably induces postoperative ileus. The transit throughout the gastrointestinal tract was investigated over several days. Digestive transit partially resumed after 96 h (Figure 2). A greater accumulation of feces was observed in the cages of mice that underwent intestinal manipulation, and the charcoal traveled a greater distance along the small intestine 96 h after the IM10′ procedure. However, gastric emptying has not yet recovered (Figure 2C). This observation confirmed that intestinal manipulation during 10 min induced a pathological ileus.

### 3.2 Anastomosis Is Not Required to Induce Postoperative Ileus in Mice

**FIGURE 3:**
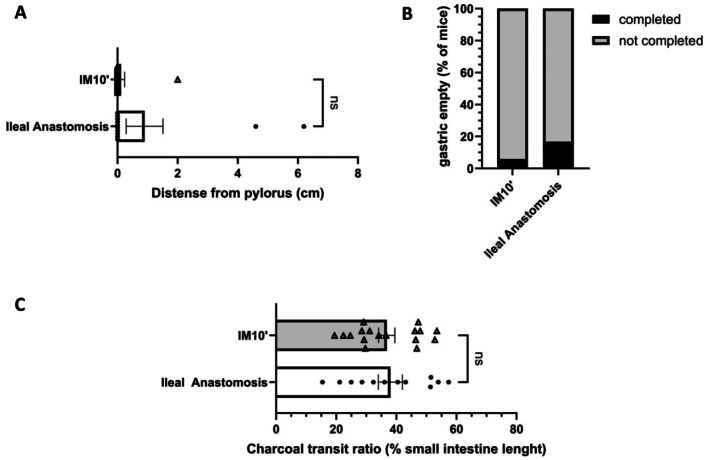
IM10′ protocol provoked a defect of gut transit comparable to anastomosis. Gastric emptying (A), gastric emptying completion (B) and small bowel transit (C) were measured in mice 24 h after ileal anastomosis or intestinal manipulation for 10 min (IM10′) (*n* = 12–17 per group). Data are presented as individual values with means and SEM. Significance levels were determined using the Mann–Whitney test: Ns (*p* > 0.05); *p < 0.05.

In order to be as close as possible to the current practice of digestive surgery in humans, we performed manipulations with anastomosis. After segmental resection of the intestine, anastomosis is required to maintain digestive continuity. Therefore, to assess the relevance of our model to clinical practices, we performed an ileo‐ileal anastomosis model and compared it to the previously described IM10′ procedure. After anastomosis of the ileum, no gastric emptying was observed in most of mice, and the intestinal transit was also drastically reduced (Figure 3). The charcoal covered the same distance along the small intestine in mice from the ileo‐ileal anastomosis group as in the IM10′ group 24 h post‐surgery. These data demonstrate that anastomosis is not necessary for the induction of postoperative ileus. In addition, they indicate that the simple gesture of gently manipulating the small intestine for 10 min is enough to stop intestinal transit, as observed in ileus after intestinal surgery to remove a lesion.

### 3.3 Intestinal Manipulation to Induce Ileus: Ex Vivo Functional Characterization

**FIGURE 4:**
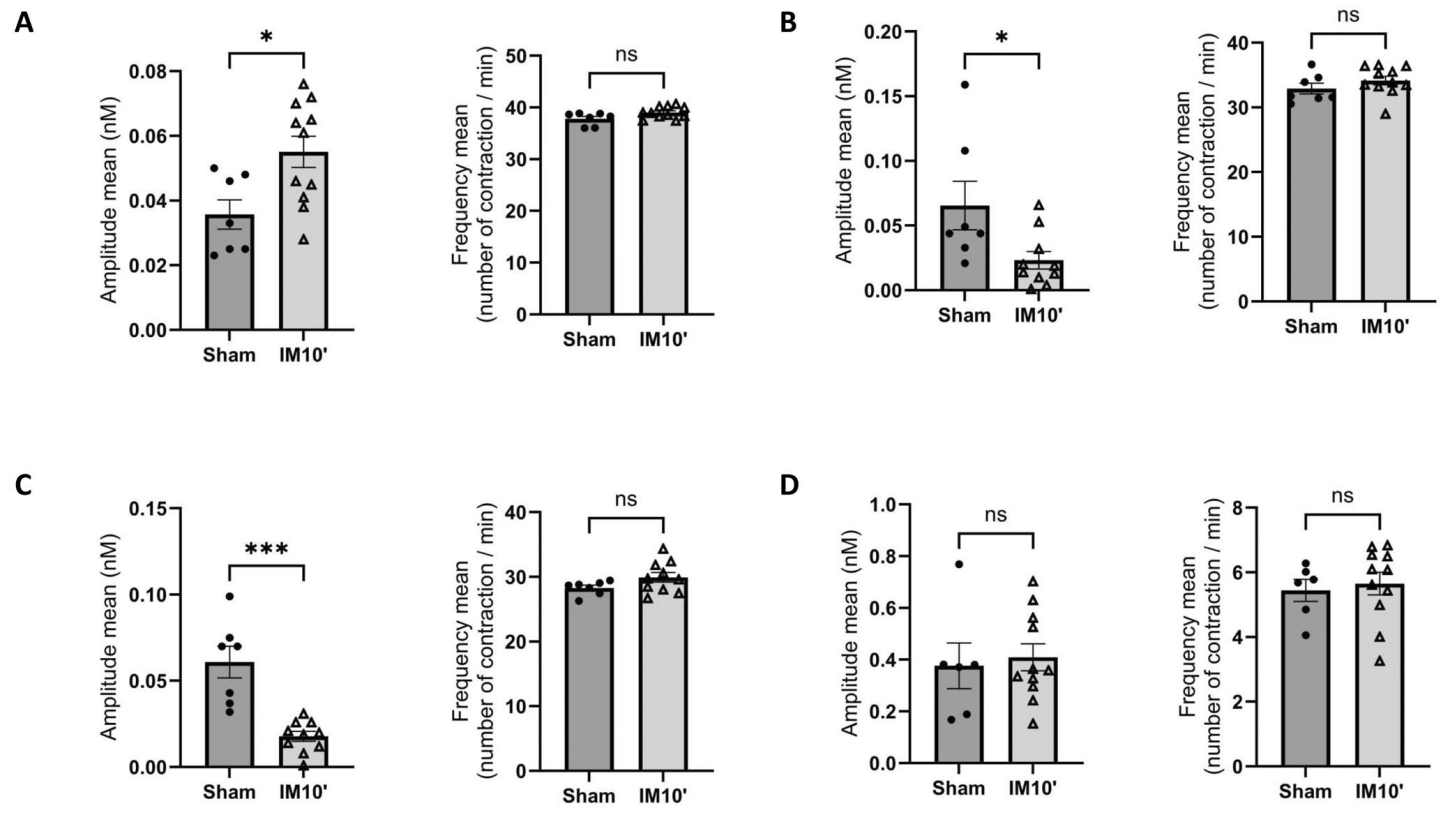
IM10′ protocol affects small intestine contractility without impacting colonic contractility. The amplitude and frequency of the contractile response of the longitudinal muscle segment were recorded from an isolated duodenum (A), jejunum (B), ileum (C) and colon (D) from mice 24 h after laparotomy (Sham) or intestinal manipulation for 10 min (IM10′) (*n* = 7–11 per group). Data are presented as individual values with means and SEM. Significance levels were determined using the Mann–Whitney test: Ns (*p* > 0.05); *p < 0.05; ***p < 0.001.

To assess intestinal motility in the different segments of the small intestine and the colon, the spontaneous contractions of three parts of the small intestine (duodenum, jejunum and ileum) and the colon were measured ex vivo. This was done using isotonic sensors to analyze both the amplitude and frequency of contractions of intestinal parts from mice subjected to sham or intestinal manipulation for 10 min to 24 h after surgery. Regarding the manipulated segment (jejunum and ileum), the contraction amplitude is decreased without affecting the frequency within the IM10′ group compared to the sham group (Figure 4B,C). In contrast, the amplitude of duodenal contractions was increased 24 h after 10 min of intestinal manipulation, but the frequency was not different compared to the sham group (Figure 4A). At the colonic level, no difference in contraction amplitude or frequency was observed within the IM10′ group compared to the sham mice (Figure 4D). These data suggest that the alteration of motility after intestinal manipulation is localized to the stomach and small intestine without affecting colonic motility.

### 3.4 Cellular and Molecular Characterization of the Inflammatory Status in POI Mice Model

**FIGURE 5:**
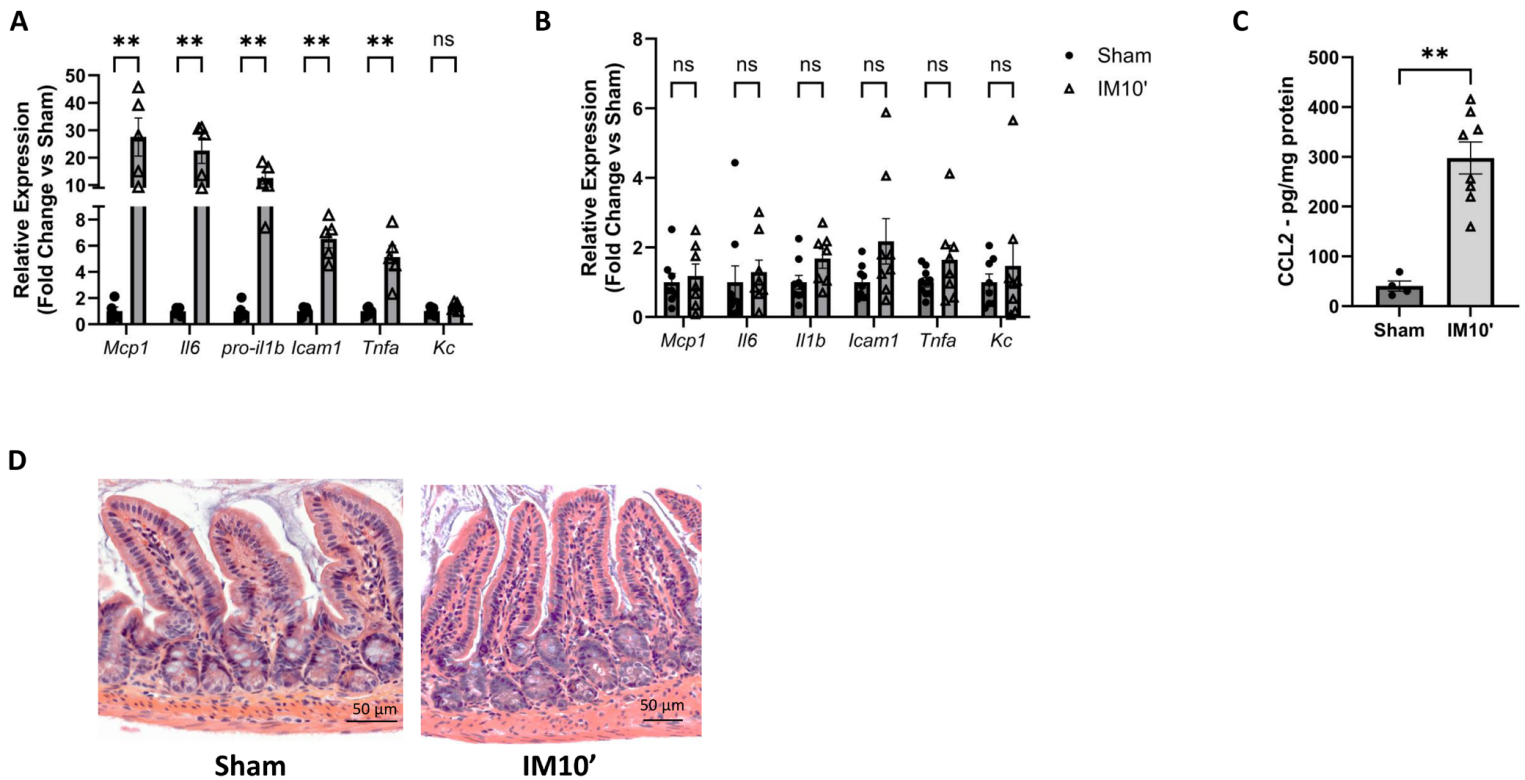
Characterization of inflammatory status in ileum after intestinal manipulation for 10 min. Relative expression of pro‐inflammatory mediators (*Mcp1*, *Il6*, *Il1b*, *Icam1*, *Tnf*α, and *Kc*) in the ileal *muscularis externa* (A) or in the ileum mucosa (B) 2.5 h post laparotomy (Sham) or small intestinal manipulation (IM10′) (*n* = 5–8). Measurement of CCL2 levels in the muscular layer (*n* = 4–8) (C) and Hematoxylin and eosin staining of the ileum 24 h post‐surgery in Sham or IM10′ mice (D). Data are presented as individual values with means and SEM. Significance levels were determined using the Mann–Whitney test: Ns (*p* > 0.05); **p < 0.01; ***p < 0.001.

Postoperative ileus entails a significant inflammatory phase primarily localized in the muscular layer of the human intestine [10]. To delineate inflammation in mice subjected to intestinal manipulation, we conducted a qPCR analysis of inflammatory mediators 2.5 h post‐surgery in the ileal muscular layer. Our results revealed a marked increase in pro‐inflammatory mediators in the IM10′ group compared to the sham group (*Mcp1, IL6, IL1b, TNFa, Icam1*), whereas *KC* analysis showed no significant difference (Figure 5A). To validate the elevation of proinflammatory mediators in the ileum muscular layers, MCP‐1/CCL2 levels were assessed via ELISA assay. MCP‐1/CCL2 demonstrated an increased level in the ileal muscular layer of IM10′ mice compared to sham mice 24 h post‐surgery (Figure 5C). Conversely, at the level of the colonic muscular layer, no significant increase in pro‐inflammatory mediators was observed (*Mcp1, IL6, IL1b, TNFa, Icam1*) following intestinal manipulation compared to sham mice (Figure S2A).

These findings indicate that inflammation is indeed present in the muscular layer of the manipulated section, whereas the colonic level appears to remain unaffected.

In addition, comparative analysis of pro‐inflammatory mediators conducted in the mucosal layer of the ileum from mice of the IM10′ group showed that the transcriptional level of pro‐inflammatory mediators was unchanged compared to mucosae from the sham group (Figure 5B). These data indicate that inflammation is preferentially localized within the muscular layer of the ileum after intestinal manipulation, as it was observed in POI patients [10].

A histological analysis was conducted to observe tissue alterations, as previously described in a rat model of postoperative ileus [14]. Histological analysis using hematoxylin and eosin (HE) staining revealed no alterations in intestinal villous architecture 24 h after intestinal manipulation. The architecture appeared similar between the intestine of sham‐operated and manipulated‐intestine mice (Figure 5D and Figure S2B). Intestinal manipulation and associated inflammation localized within the muscular layer are not associated with major histological alterations.

**FIGURE 6:**
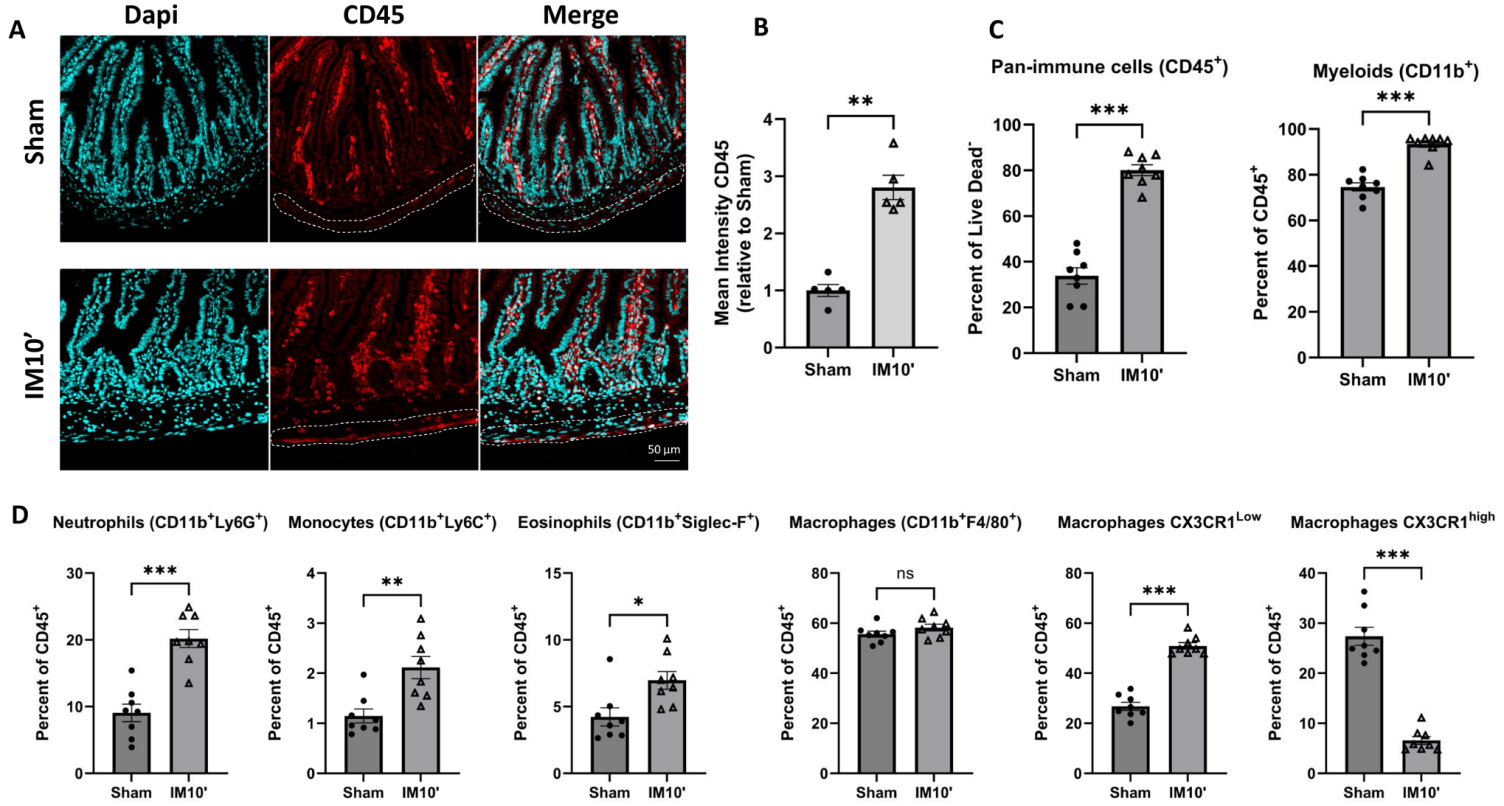
Characterization of inflammatory immune cell recruitment in ileal muscularis externa after intestinal manipulation for 10 min. Representative images of nuclear staining (blue, DAPI), pan‐immune cell staining (red, CD45) and merged images from sham and IM10′ mice (A). Quantification of CD45 positive signal (B) on immunostaining images (*n* = 5 per group). Immune cell recruitment characterized by flow cytometry (C, D) 24 h after chirurgical procedure in the ileal muscularis externa from Sham (laparotomy) or IM10′ (small intestinal manipulation for 10 min) mice (*n* = 8 per group). Data are presented as individual values with mean ± SEM. Significance levels were determined using the Mann–Whitney test: Ns (*p* > 0.05); *p < 0.05; **p < 0.01; ***p < 0.001.

However, POI was previously characterized by an influx of immune cells into the muscular layer of the intestine [15, 16]. To assess this influx in our mice model, pan‐leukocyte (CD45) staining was conducted via immunofluorescence at the ileum level 24 h after surgery. A higher CD45 signal was observed in the muscular layer of the ileum from mice of the IM10′ group compared to the sham group (Figure 6A,B), indicating the presence of an influx of immune cells. In the colon, where no upregulation of inflammatory markers was detected, there was also no observed influx of CD45+ cells (Figure S2C). Furthermore, to characterize the leukocyte influx 24 h after intestinal manipulation, an analysis of myeloid subpopulations was performed by flow cytometry within the muscular layer of the ileum. We observed an increase of immune cells with a majority of myeloid cells (Figure 6C). Also, there was an increase of neutrophils, monocytes, and eosinophils into the external muscularis, while total macrophages showed no significant change (Figure 6D). However, there was an increase in monocyte‐derived macrophages (expressing low level of CX3CR1) and a decrease in resident macrophages (expressing high level of CX3CR1) (Figure 6D and Figure S3). Overall, these results indicate that 24 h after intestinal manipulation for 10 min, inflammation was sustained by an influx of neutrophils, monocytes, and monocyte‐derived macrophages.

## 4 Discussion

Postoperative ileus is an iatrogenic complication that occurs after abdominal surgery. The cessation of intestinal transit associated with inflammation is characteristic of POI [1, 17]. Today, no pharmacological molecule has shown efficacy in the prevention or treatment of pathologic POI [5]. The development of a mouse model is therefore essential in order to understand the mechanisms or to test pharmacological molecules for the treatment of postoperative ileus. The literature described two mouse models of POI. The first, described by Vilz et al., consists of externalizing the small intestine and evacuating all the luminal contents present from the pylorus to the caecum using two cotton swabs with simultaneous rolling action on the small intestine [18]. This model has a number of limitations, notably significant histological lesions of mucosae that were induced [14]. Moreover, the compression action of cotton swabs is a gesture very different from that performed by surgeons during digestive surgery. It could therefore lead to the activation of mechanisms not involved in human pathology.

The second murine model of POI has been described by Van Bree et al. This protocol is a robust procedure to induce POI in mice. However, it requires a purpose‐designed instrument that allows constant pressure to be applied to the small intestine for a short time [19]. This specific equipment is manufactured in‐house, and the procedure is far removed from the surgeon's movements. In this context, it is necessary to develop a murine model of postoperative ileus that is accessible and closer to human abdominal surgery.

Colonic anastomosis, particularly within the setting of colorectal surgery, is among the procedures most frequently associated with severe postoperative ileus [20]. In mice, the procedure proved technically challenging due to the fragility of the murine colon. The sutures were unable to maintain tissue integrity, leading to rapid mortality in all animals. We therefore performed intestinal manipulation and ileal anastomosis on mice. This procedure stopped intestinal transit and prevented gastric emptying. Considering that this procedure is long and delicate, we developed a mice model of ileus which mimics it regarding the defect of intestinal transit. Understanding the pathophysiological pathways of POI needs to be based on a simple and robust mice model. The externalization and manipulation of the small intestine for 10 min, such as when the surgeon is looking for a lesion or removing an area that is too badly damaged, induced a delay in gastrointestinal transit and absence of gastric emptying. In addition, despite the fact that at least three persons performed the IM10′ procedure, the dispersion of data around the mean was significantly smaller in the group of mice subjected to 10 min of handling compared to the other experimental groups, for both the speed of food bolus movement and gastric emptying. After this procedure, all mice demonstrated a total absence of gastric emptying; the gastric peristalsis stopped as observed after anastomosis. The food bolus covered < 40% of the total length of the small intestine in 20 min. Propulsion is halved compared with the sham group. In accordance with what is observed in patients, the delay in intestinal transit is associated with dilation of duodenum [11]. Precise analysis showed that 24 h after the intestinal manipulation, the peristaltism of the small intestine is affected by the procedure. Interestingly, the two parts of the small intestine showed opposite phenotypes, with the amplitude of contractions in the manipulated segment reduced, in contrast to the increased amplitude of contractions in the duodenum, which was not manipulated due to the mesenteric folds between the duodenum and colon in mice. It has been shown that there is a correlation between the duodenal dilation and a relaxation of the stomach contractions, suggesting that the duodenal dilation could be responsible for the gastroparesis leading to the stasis of food content [21, 22]. In addition, some authors showed a negative relationship between the duodenal activity and gastric emptying, a high duodenal contractile activity leading to a slower gastric empty [23].

Ten minutes of intestinal manipulation is sufficient to induce a severe ileus. Indeed, first feces were observed only after more than 3 days after intestinal manipulation, and gastric emptying was still ineffective. This observation is in accordance with what is observed in the pig model [24] and in patients suffering from morbid POI [25].

Several studies have shown that immune cell recruitment is responsible for impaired intestinal transit [16, 26]. In the present model, a massive influx of monocytes, neutrophils, and immature macrophages was characterized in the muscularis 24 h after intestinal manipulation. As very well described by Farro et al. in another model of POI, 10 min of manipulation led to upregulation of pro‐inflammatory cytokines as the first event leading to the recruitment of immune cells in the muscularis from the bloodstream. This inflammatory response is restricted to this layer of intestinal tissue as no transcriptional variation was observed higher in the mucosae. In addition, no histological abnormalities were observed except for this immune infiltrate in the muscularis.

In conclusion, these data have enabled us to show that the IM10′ procedure enables inducing postoperative ileus in mice in a very simple and reproducible way, mimicking the surgeon's actions in human practice. The intestinal transit defects induced by this procedure are identical to those resulting from anastomosis. This IM10′ model presents all the characteristics already described for postoperative ileus, that is, impaired gastric emptying and small intestinal transit, localized inflammation in the external intestinal muscularis characterized by an increase in pro‐inflammatory mediators and an influx of immune cells.

Therefore, the ‘IM10’ model is a very simple robust procedure that does not require any specific equipment and mimics clinical practice, making it possible to characterize the underlying mechanisms or to test pharmacological molecules for the treatment of postoperative ileus.

## Author Contributions

C.D., E.B., and N.V. conceived the study. C.D. and E.B. supervised the project. R.G., J.T., T.P., T.B., P.R., and S.W. performed the experiments and collected the data. A.C. carried out the imaging analyses. C.K. contributed to the isolated organ analyses. R.G., E.B., and C.D. drafted the first version of the manuscript. C.D. performed the review and editing. All authors read and approved the final version of the manuscript.

## Conflicts of Interest

The authors declare no conflicts of interest.

## Supporting information


**Data S1:** nmo70157‐sup‐0001‐supinfo.pdf.

## Data Availability

The data that support the findings of this study are available from the corresponding author upon reasonable request.

## References

[1] E. Buscail and C. Deraison , “Postoperative Ileus: A Pharmacological Perspective,” British Journal of Pharmacology 179, no. 13 (2022): 3283–3305, 10.1111/bph.15800. 35048360

[2] S. Solanki , R. C. Chakinala , K. F. Haq , et al., “Paralytic Ileus in the United States: A Cross‐Sectional Study From the National Inpatient Sample,” SAGE Open Medicine 8 (2020): 2050312120962636, 10.1177/2050312120962636. 33088567 PMC7545785

[3] A. Venara , M. Neunlist , K. Slim , et al., “Postoperative Ileus: Pathophysiology, Incidence, and Prevention,” Journal of Visceral Surgery 153, no. 6 (2016): 439–446, 10.1016/j.jviscsurg.2016.08.010. 27666979

[4] L. Traeger , M. Koullouros , S. Bedrikovetski , H. M. Kroon , J. W. Moore , and T. Sammour , “Global Cost of Postoperative Ileus Following Abdominal Surgery: Meta‐Analysis,” BJS Open 7, no. 3 (2023): zrad054, 10.1093/bjsopen/zrad054. 37352872 PMC10289829

[5] E. Buscail , T. Planchamp , G. Le Cosquer , et al., “Postoperative Ileus After Digestive Surgery: Network Meta‐Analysis of Pharmacological Intervention,” British Journal of Clinical Pharmacology 90, no. 1 (2024): 107–126, 10.1111/bcp.15878. 37559444

[6] G. E. Boeckxstaens and W. J. de Jonge , “Neuroimmune Mechanisms in Postoperative Ileus,” Gut 58, no. 9 (2009): 1300–1311, 10.1136/gut.2008.169250. 19671558

[7] G. E. Boeckxstaens , D. P. Hirsch , A. Kodde , et al., “Activation of an Adrenergic and Vagally‐Mediated NANC Pathway in Surgery‐Induced Fundic Relaxation in the Rat,” Neurogastroenterology and Motility 11, no. 6 (1999): 467–474, 10.1046/j.1365-2982.1999.00172.x. 10583854

[8] B. Y. De Winter , G. E. Boeckxstaens , J. G. De Man , T. G. Moreels , A. G. Herman , and P. A. Pelckmans , “Effect of Adrenergic and Nitrergic Blockade on Experimental Ileus in Rats,” British Journal of Pharmacology 120, no. 3 (1997): 464–468, 10.1038/sj.bjp.0700913. 9031750 PMC1564477

[9] G. Farro , P. J. Gomez‐Pinilla , M. Di Giovangiulio , et al., “Smooth Muscle and Neural Dysfunction Contribute to Different Phases of Murine Postoperative Ileus,” Neurogastroenterology and Motility 28, no. 6 (2016): 934–947, 10.1111/nmo.12796. 26891411

[10] F. O. The , R. J. Bennink , W. M. Ankum , et al., “Intestinal Handling‐Induced Mast Cell Activation and Inflammation in Human Postoperative Ileus,” Gut 57, no. 1 (2008): 33–40, 10.1136/gut.2007.120238. 17591620

[11] T. L. Hedrick , M. D. McEvoy , M. (M.) G. Mythen , et al., “American Society for Enhanced Recovery and Perioperative Quality Initiative Joint Consensus Statement on Postoperative Gastrointestinal Dysfunction Within an Enhanced Recovery Pathway for Elective Colorectal Surgery,” Anesthesia and Analgesia 126, no. 6 (2018): 1896, 10.1213/ANE.0000000000002742. 29293183

[12] T. Ahrends , M. Weiner , and D. Mucida , “Isolation of Myenteric and Submucosal Plexus From Mouse Gastrointestinal Tract and Subsequent Flow Cytometry and Immunofluorescence,” STAR Protocols 3, no. 1 (2022): 101157, 10.1016/j.xpro.2022.101157. 35146454 PMC8819479

[13] B. Weigmann , I. Tubbe , D. Seidel , A. Nicolaev , C. Becker , and M. F. Neurath , “Isolation and Subsequent Analysis of Murine Lamina Propria Mononuclear Cells From Colonic Tissue,” Nature Protocols 2, no. 10 (2007): 2307–2311, 10.1038/nprot.2007.315. 17947970

[14] J. C. Kalff , W. H. Schraut , R. L. Simmons , and A. J. Bauer , “Surgical Manipulation of the Gut Elicits an Intestinal Muscularis Inflammatory Response Resulting in Postsurgical Ileus,” Annals of Surgery 228, no. 5 (1998): 652–663. 9833803 10.1097/00000658-199811000-00004 PMC1191570

[15] G. Farro , M. Stakenborg , P. J. Gomez‐Pinilla , et al., “CCR2‐Dependent Monocyte‐Derived Macrophages Resolve Inflammation and Restore Gut Motility in Postoperative Ileus,” Gut 66, no. 12 (2017): 2098–2109, 10.1136/gutjnl-2016-313144. 28615302

[16] J. C. Kalff , T. M. Carlos , W. H. Schraut , T. R. Billiar , R. L. Simmons , and A. J. Bauer , “Surgically Induced Leukocytic Infiltrates Within the Rat Intestinal Muscularis Mediate Postoperative Ileus,” Gastroenterology 117, no. 2 (1999): 378–387, 10.1053/gast.1999.0029900378. 10419919

[17] N. Stakenborg , E. Labeeuw , P. J. Gomez‐Pinilla , et al., “Preoperative Administration of the 5‐HT4 Receptor Agonist Prucalopride Reduces Intestinal Inflammation and Shortens Postoperative Ileus via Cholinergic Enteric Neurons,” Gut 68, no. 8 (2019): 1406–1416, 10.1136/gutjnl-2018-317263. 30472681 PMC6691854

[18] T. O. Vilz , M. Overhaus , B. Stoffels , M. von Websky , J. C. Kalff , and S. Wehner , “Functional Assessment of Intestinal Motility and Gut Wall Inflammation in Rodents: Analyses in a Standardized Model of Intestinal Manipulation,” Journal of Visualized Experiments 11, no. 67 (2012): 4086, 10.3791/4086. PMC3490263 22990580

[19] S. H. van Bree , A. Nemethova , F. S. van Bovenkamp , et al., “Novel Method for Studying Postoperative Ileus in Mice,” International Journal of Physiology, Pathophysiology and Pharmacology 4 (2012): 219–227. 23320135 PMC3544220

[20] P. H. Chapuis , L. Bokey , A. Keshava , et al., “Risk Factors for Prolonged Ileus After Resection of Colorectal Cancer: An Observational Study of 2400 Consecutive Patients,” Annals of Surgery 257, no. 5 (2013): 909–915, 10.1097/SLA.0b013e318268a693. 23579542

[21] F. De Ponti , F. Azpiroz , and J. R. Malagelada , “Reflex Gastric Relaxation in Response to Distention of the Duodenum,” American Journal of Physiology‐Gastrointestinal and Liver Physiology 252, no. 5 (1987): G595–G601, 10.1152/ajpgi.1987.252.5.G595. 2883897

[22] H. H. Holzer and H. E. Raybould , “Vagal and Splanchnic Sensory Pathways Mediate Inhibition of Gastric Motility Induced by Duodenal Distension,” American Journal of Physiology‐Gastrointestinal and Liver Physiology 262, no. 4 (1992): G603–G608, 10.1152/ajpgi.1992.262.4.G603. 1566842

[23] N. W. Weisbrodt , J. N. Wiley , B. F. Overholt , and P. Bass , “A Relation Between Gastroduodenal Muscle Contractions and Gastric Empyting,” Gut 10, no. 7 (1969): 543–548, 10.1136/gut.10.7.543. 5806934 PMC1552942

[24] A. K. Martensen , E. V. Moen , C. Brock , and J. A. Funder , “Postoperative Ileus—Establishing a Porcine Model,” Neurogastroenterology and Motility 36, no. 9 (2024): e14872, 10.1111/nmo.14872. 39138548

[25] S. H. W. Van Bree , W. A. Bemelman , M. W. Hollmann , et al., “Identification of Clinical Outcome Measures for Recovery of Gastrointestinal Motility in Postoperative Ileus,” Annals of Surgery 259, no. 4 (2014): 708–714, 10.1097/SLA.0b013e318293ee55. 23657087

[26] J. C. Kalff , B. M. Buchholz , M. K. Eskandari , et al., “Biphasic Response to Gut Manipulation and Temporal Correlation of Cellular Infiltrates and Muscle Dysfunction in Rat,” Surgery 126, no. 3 (1999): 498–509, 10.1016/S0039-6060(99)70091-7. 10486602

