# Supplementary material for: Modeling Postoperative Pathologic Ileus in Mice: A Simplified and Translational Approach
Source: Neurogastroenterol Motil. 2025 Sep 22;37(11):e70157. doi: 10.1111/nmo.70157 (PMC12534576; doi:10.1111/nmo.70157)
Supplement: Supplementary file 1 — Data S1: nmo70157‐sup‐0001‐supinfo.pdf. [file NMO-37-e70157-s001.pdf]

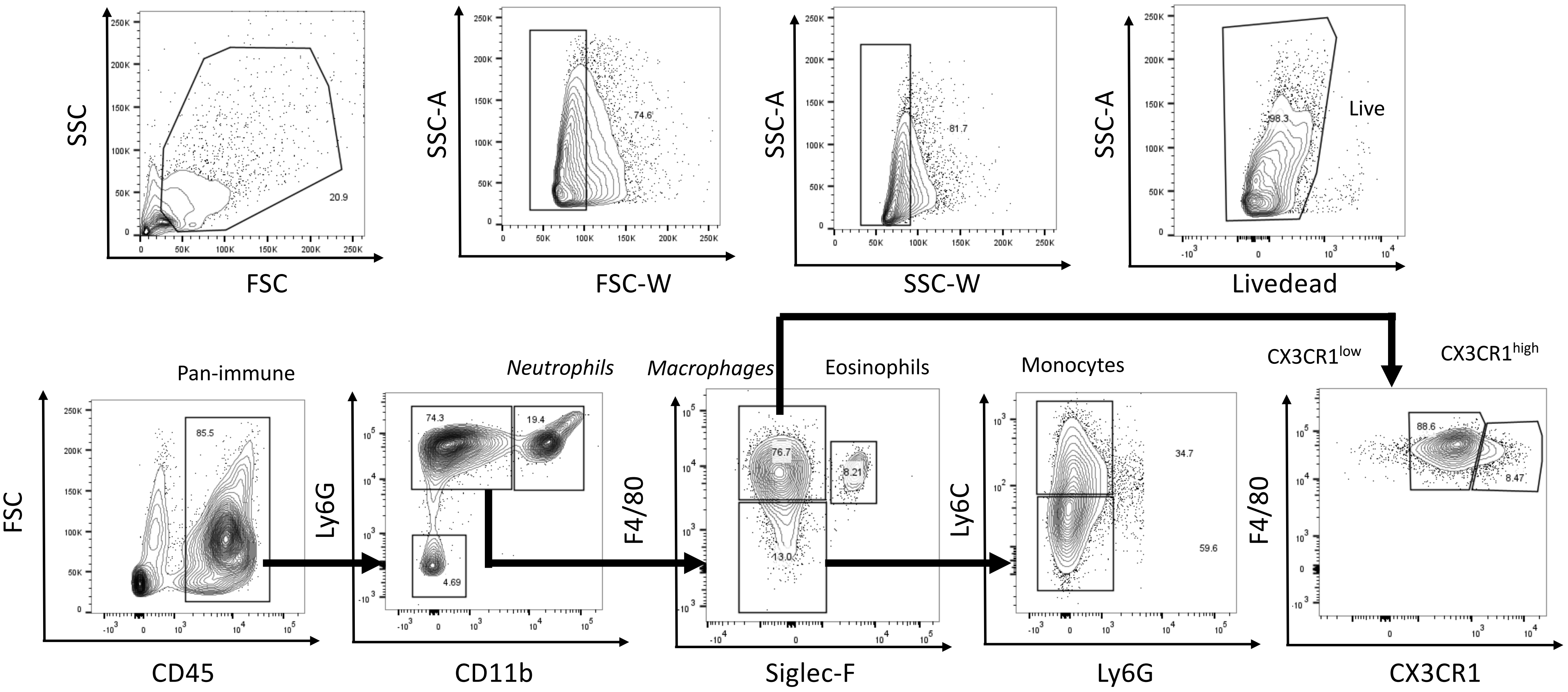

Figure S1 : Flow cytometry gating strategy for muscular layers of the ileum
Flow cytometry gating strategy used to identify Macrophages, Eosinophils, Monocytes, Neutrophils and Macrophage CX3CR1^{low} or CX3CR1^{high}.

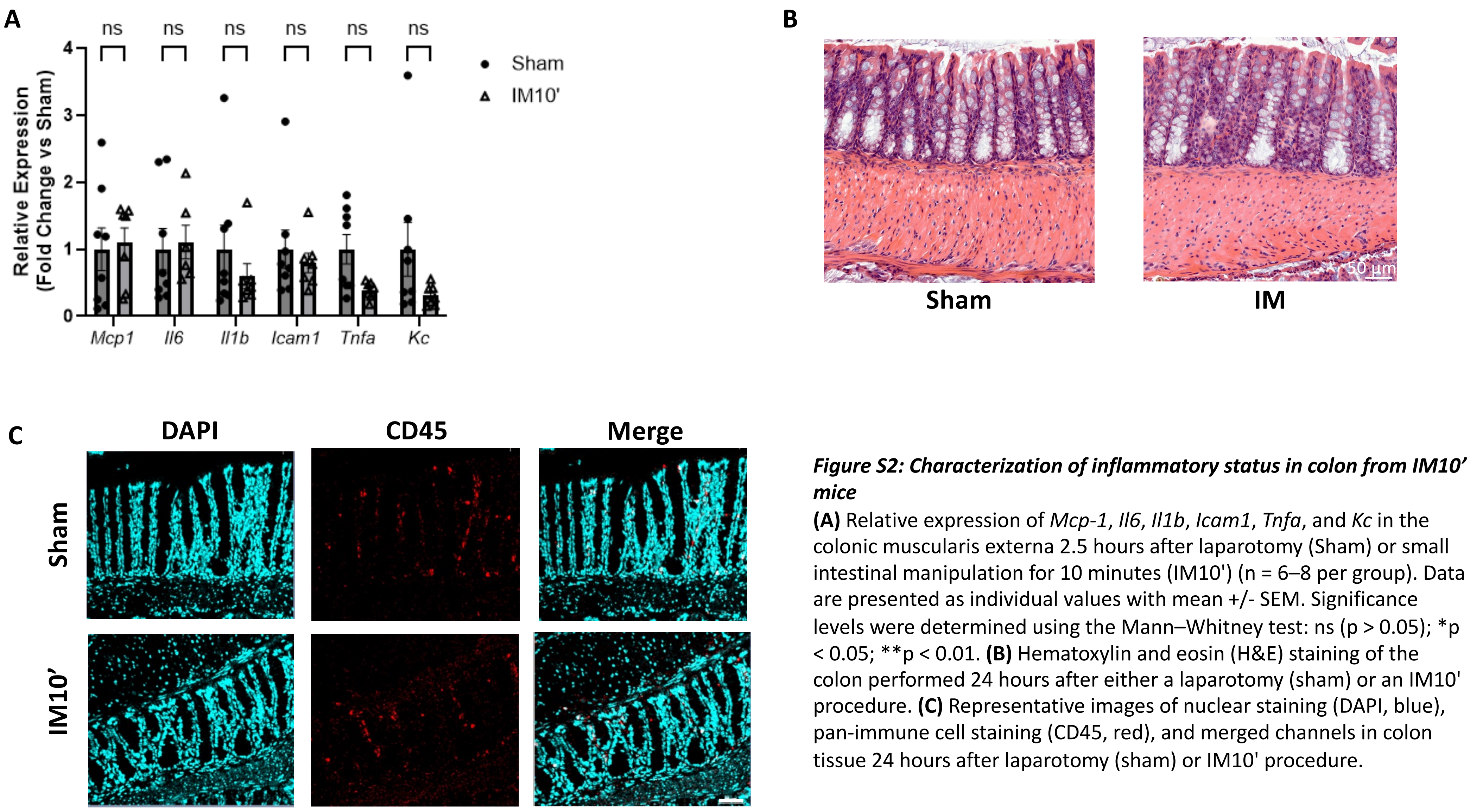

Figure S2: Characterization of inflammatory status in colon from IM10' mice

(A) Relative expression of *Mcp-1*, *Il6*, *Il1b*, *Icam1*, *Tnfa*, and *Kc* in the colonic muscularis externa 2.5 hours after laparotomy (Sham) or small intestinal manipulation for 10 minutes (IM10') (n = 6–8 per group). Data are presented as individual values with mean +/- SEM. Significance levels were determined using the Mann–Whitney test: ns ($p > 0.05$); *$p < 0.05$; **$p < 0.01$. **(B)** Hematoxylin and eosin (H&E) staining of the colon performed 24 hours after either a laparotomy (sham) or an IM10' procedure. **(C)** Representative images of nuclear staining (DAPI, blue), pan-immune cell staining (CD45, red), and merged channels in colon tissue 24 hours after laparotomy (sham) or IM10' procedure.

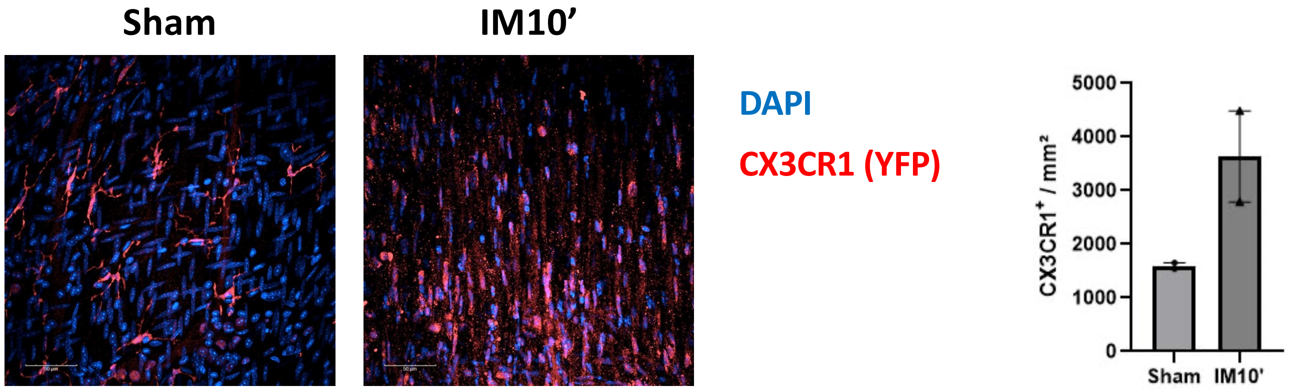

Figure S3 :Immunodetection of CX3CR1^{+} cells in ileal longitudinal muscle myenteric plexus.
Representative images of nuclear staining (DAPI, blue) and YFP staining (red) from sham and IM10’ CX3CR1 transgenic mice (n = 2 per group).

Table S1: Overview of the different surgical procedures

Surgical Procedure	Abreviation	Description	Number of mice per groupe (=n)
**Steady state mice**	Basal	Mice with no anesthesia or surgery	10
**Midline laparotomy**	Sham	Mice subjected to anesthesia, to laparotomy without externlisation of intestine, and analgesia	30
**Small Intestine Externalization**	SI Ext.	Mice subjected to anesthesia, to laparotomy with small intestine externalization, and analgesia	27
**Small Intestine and caecum externalization**	SI + caecum Ext.	Mice subjected to anesthesia, to laparotomy with small intestine and ceacum externalization, and analgesia	6
**Small Intestine and caecum externalization, Intestinale manipulation 2 min**	SI + caecum Ext. + IM2'	Mice subjected to anesthesia, to laparotomy with small intestine and ceacum externalization, and analgesia<br>Small intestine and ceacum were manipulated for 2 min	11
**Small Intestine and caecum externalization, Intestinale manipulation 10 min**	SI + caecum Ext. + IM10'	Mice subjected to anesthesia, to laparotomy with small intestine and ceacum externalization, and analgesia<br>Small intestine and ceacum were manipulated for 10 min	24

Table S2: Motility parameters in different surgical procedures.

Surgical Procedure	Abreviation	Gastric emptying (Mean +/- SEM)	Small intestine transit (Mean +/- SEM)	Number of mice per groupe (=n)
**Steady state mice**	Basal	7,98 +/- 0,63	70,96 +/- 3,24	10
**Midline laparotomy**	Sham	5,953 +/- 0,69*, #	67,39 +/- 2,93	30
**Small Intestine Externalization**	SI Ext.	2,459 +/- 0,61*, #	50,6 +/- 3,49*, #	27
**Small Intestine and caecum externalization**	SI + caecum Ext.	2,28 +/- 1,46*, #	57,02 +/- 4,26	6
**Small Intestine and caecum externalization, Intestinale manipulation 2 min**	SI + caecum Ext. + IM2'	3,573 +/- 1,33*, #	66,74 +/- 5,16	11
**Small Intestine and caecum externalization, Intestinale manipulation 10 min**	SI + caecum Ext. + IM10'	0,08333 +/- 0,09*, #	35,53 +/- 2,54*, #	24

Note: * p< 0.05 compared to basal condition, # p< 0.05 compared to sham condition
